# Editorial: Factors associated with drug resistance and virulence of *Mycobacterium tuberculosis*

**DOI:** 10.3389/fcimb.2024.1504923

**Published:** 2024-12-19

**Authors:** Natalie Eva Nieuwenhuizen, Lei Ji

**Affiliations:** ^1^ Institute of Hygiene and Microbiology, Julius Maximilian University of Würzburg, Würzburg, Germany; ^2^ School of Basic Medical Sciences and Forensic Medicine, Hangzhou Medical College, Hangzhou, Zhejiang, China

**Keywords:** Mycobacterium tuberculosis, drug resistance, tuberculosis, virulence, antibiotics

Tuberculosis (TB), caused by the bacillus *Mycobacterium tuberculosis* (*Mtb*), remains a leading cause of death by infectious disease worldwide, with over 10 million people infected annually (World Health Organization, 2023). The emergence of drug-resistant *Mtb* strains has increased the duration, cost, toxicity and difficulty of TB therapy, and is a major public health concern. The objective of this Research Topic was to highlight some recent advancements in the understanding of drug resistance and virulence of *Mtb*. For instance, Liu et al. investigated how Rv1987 influences the lung microbiota and immune responses in mice, and how these interactions contributed to the survival of *Mtb*.

The standard treatment regime for drug-sensitive TB is two months of therapy with rifampicin (RIF), isoniazid (INH), pyrazinamide (PZA), and ethambutol (EMB), followed by four months treatment with RIF and INH (World Health Organization, 2022) Infection with *Mtb* strains resistant to RIF and INH is termed multidrug-resistant TB (MDR-TB), but RIF-mono-resistant TB is routinely treated the same as MDR-TB (Lange et al., 2019). As part of this Research Topic, Birhanu et al. performed a meta-analysis on the prevalence of RIF-resistant *Mtb* in Ethiopia and found it was around 7%. Worldwide, around 5% of patients with TB have MDR-TB, but in some countries (Moldova, Ukraine, Kazakhstan, Kyrgyzstan) the proportion exceeds 25%. The starting treatment regimen for TB should consist of at least four drugs that are likely to be active (Lange et al., 2019; World Health Organization, 2019). The WHO recommends including fluoroquinolones (FQs), bedaquiline (BDQ), and linezolid (LZD), as they are considered highly effective (34). TB caused by an *Mtb* strain which is resistant to INH, RIF and a FQ is defined as pre-extensively drug resistant TB (pre-XDR TB), while XDR TB is defined as TB caused by strains that are resistant to INH, RIF, a FQ, and either BDQ or LZD or both (World Health Organization, 2019) (WHO). BDQ, delamanid (DLM) and pretomanid (PTM) were the first novel TB drugs to be developed after a gap of four decades (Bloemberg et al., 2015; Dookie et al., 2022). The remarkable efficacy of BDQ and PTM improved treatment success and led to the introduction of a 6-month short-course regimen for MDR-TB (Dookie et al., 2022). Unfortunately, within years of the introduction of these novel drugs, the first clinical isolates of *Mtb* resistant to BDQ and DLM were identified (Bloemberg et al., 2015). The rapid appearance of BDQ-resistant strains is disturbing. Interestingly, there is cross-resistance between BDQ and CFZ due to their shared efflux pathways, indicating that the use of CFZ could lead to BDQ resistance (Ismail et al.). As part of this Research Topic, Islam et al. reviewed recent studies on the mechanisms of action of BDQ and CFZ, individual resistance and cross-resistance.

Antimicrobial resistance can be intrinsic, acquired, or adaptive (Fernandez et al., 2011; Crimi et al., 2020). The formation of biofilms by *Mtb* is an important intrinsic factor in drug resistance, since this physical barrier protects the bacilli against antibiotics (Esteban and Garcia-Coca, 2017). Furthermore, *Mtb* is intrinsically resistant to many antibiotics due to its secretion of drug-modifying and degrading enzymes and its unique cell envelope, composed of long-chain mycolic acids, highly branched arabinogalactan polysaccharides and a meshwork of cross-linked, modified peptidoglycans (Gygli et al., 2017). For example, *Mtb* is resistant to most beta-lactams due to its expression of beta-lactamase, the impermeable nature of its cell wall and its non-classical peptidoglycan cross-links (Catalao et al.). Some of the distinctive modifications to the peptidoglycan layer are carried out by enzymes encoded by *namH* and *murT/gatD* (Raymond et al., 2005; Catalao et al.). Silveiro et al. used CRISPR interference to silence these genes in the model organism *M. smegmatis*, and found that amidation of D-iso-glutamate played a role in cefotaxime and isoniazid resistance while N-glycolylation of D-iso-glutamate promoted resistance to beta-lactams. Thus, drugs targeting peptidoglycan modifications may be a useful avenue for developing novel TB therapies.

Acquired resistance to antibiotics in bacteria occurs as a result of chromosomal genetic mutations or transfer of mobile genetic elements (Gygli et al., 2017). The vast majority of drug resistant phenotypes in *Mtb* are due to chromosomal mutations, since there appears to be a lack of horizontal gene transfer in this species. Genetic mutations that bestow resistance include those that lead to the overexpression or alteration of the drug target, overexpression of efflux pumps or abrogation of prodrug activation. Advances in our understanding of nucleic acid biology have further revealed that adaptive epigenetic mechanisms can also contribute to drug resistance in bacteria (Crimi et al., 2020). Epigenetic changes can affect gene expression without altering the DNA sequences and include changes in histone modification, DNA methylation, and expression of non-coding RNA molecules. One of the main reasons for TB relapse, drug resistance and the necessity for long therapy is the presence of bacteria known as persisters (Zhang et al., 2012). After being taken up by macrophages, *Mtb* bacilli can enter a persistent state, in which they are dormant, which protects them from killing by antibiotics. Persisters are genetically identical to the rest of the bacterial population. It has been shown that *Mtb*-induced epigenetic changes can play an important role in persistence by promoting its survival in the host immune cells (Marimani et al., 2018). Consequently, it has been suggested that combinatorial therapy using conventional TB drugs and agents targeting *Mtb*-driven epigenetic changes may improve TB treatment and reduce drug resistance (Marimani et al., 2018; Crimi et al., 2020).

In bacteria, DNA methylation is the main epigenetic mechanism for the regulation of gene expression (Gao et al., 2023). Wu et al. set out to investigate the epigenetic features associated with resistance to EMB by inducing mono-EMB resistant *Mtb in vitro*, using a multi-omics approach. Fifteen genes with high methylation and low expression were identified in EMB-resistant strains, and proteomics analysis showed that the gene products of three of these (*mbtB*, *mbtD*, and *celA1*) were significantly downregulated. Further investigations found that expression of *mbtD* and *celA* was significantly downregulated in EMB-resistant clinical strains compared to susceptible strains. Studies suggest that *celA1* inhibits biofilm formation and reduces antibiotic tolerance (Van Wyk et al., 2017; Savijoki et al., 2021), while *mbtD* encodes a polyketide synthase required for the synthesis of mycobactins, iron chelators that scavenge iron during growth within macrophages (Boeck et al., 2022). In *Mycobacterium abscessus*, *mbtD* plays a role in intracellular survival. As part of this Research Topic, Wang et al. reviewed the importance of metal ions in the survival of *Mtb* and suggest that targeting transcriptional regulatory proteins involved in metal ion regulation is a promising strategy for drug development.

Post-translational modification of proteins is another mechanism of cell adaptation to a changing environment. Such modifications include acetylation, phosphorylation, ubiquitination and pupylation. As part of this Research Topic, Huang et al. reviewed the role of acetylation by *Mtb* in its virulence, host immunity and drug resistance. Acetylation can regulation the transcription, translation, and folding of proteins. Various *Mtb* acetyltransferases have been identified and confirmed to act as virulence factors. Acetyltransferases of Mtb can modify small molecular substrates, including antibiotics, leading to resistance (Schwarz et al., 2004; Sanz-Garcia et al., 2019). For example, the acetyltransferase Rv2170 is associated with resistance to INH, since acetylated INH is readily degraded (Arun et al., 2020). Interestingly, host polymorphisms in N-acteyltransferase 2 affect the metabolic rate of INH, affecting its therapeutic effect and toxicity in different individuals (Jing et al., 2020).

## Conclusion

Drug-resistant *Mtb* strains pose a significant global health threat. This Research Topic highlights some of the recent advancements in understanding drug resistance and virulence mechanisms of *Mtb*, and suggests areas for future research. Exploring factors such as epigenetic and post-translational modifications in drug resistance may identify potential targets for novel anti-TB therapies.
